# Characterization of *Fusobacterium varium* Fv113-g1 isolated from a patient with ulcerative colitis based on complete genome sequence and transcriptome analysis

**DOI:** 10.1371/journal.pone.0189319

**Published:** 2017-12-07

**Authors:** Tsuyoshi Sekizuka, Yumiko Ogasawara, Toshifumi Ohkusa, Makoto Kuroda

**Affiliations:** 1 Laboratory of Bacterial Genomics, Pathogen Genomics Center, National Institute of Infectious Diseases, Tokyo, Japan; 2 Department of Microbiota Research, Juntendo University Graduate School of Medicine, Tokyo, Japan; Kyungpook National University, REPUBLIC OF KOREA

## Abstract

*Fusobacterium* spp. present in the oral and gut flora is carcinogenic and is associated with the risk of pancreatic and colorectal cancers. *Fusobacterium* spp. is also implicated in a broad spectrum of human pathologies, including Crohn’s disease and ulcerative colitis (UC). Here we report the complete genome sequence of *Fusobacterium varium* Fv113-g1 (genome size, 3.96 Mb) isolated from a patient with UC. Comparative genome analyses totally suggested that Fv113-g1 is basically assigned as *F*. *varium*, in particular, it could be reclassified as notable *F*. *varium* subsp. similar to *F*. *ulcerans* because of partial shared orthologs. Compared with the genome sequences of *F*. *varium* ATCC 27725 (genome size, 3.30 Mb) and other strains of *Fusobacterium* spp., Fv113-g1 possesses many accessary pan-genome sequences with noteworthy multiple virulence factors, including 44 autotransporters (type V secretion system, T5SS) and 13 *Fusobacterium* adhesion (FadA) paralogs involved in potential mucosal inflammation. Indeed, transcriptome analysis demonstrated that Fv113-g1-specific accessary genes, such as multiple T5SS and *fadA* paralogs, showed notably increased expression with D-MEM cultivation than with brain heart infusion broth. This implied that growth condition may enhance the expression of such potential virulence factors, leading to remarkable survival against other gut microorganisms and to the pathogenicity to human intestinal epithelium.

## Introduction

Fusobacteria are anaerobic gram-negative rods that rarely cause clinically significant and serious infections in humans [1]. Two *Fusobacterium* spp. *F*. *nucleatum* and *F*. *necrophorum* are the most commonly isolated pathogens within this genus. *F*. *nucleatum* is an anaerobic oral commensal that acts as an agent in gingival infection and is a periodontal pathogen [2]. Periodontal disease and dental procedures are frequently identified as the source of invasive *F*. *nucleatum* infection, which is implicated in oral infections, adverse pregnancy outcomes, GI disorders, and various other human diseases. Similarly, chemotherapy-induced oropharyngeal mucositis [3] and inflammatory bowel disease [4] have been implicated in invasive *F*. *nucleatum* infection. *F*. *necrophorum* is one of the causal agents for Lemierre’s syndrome [5,6], which is characterized by sepsis that often evolves after a sore throat or tonsillitis and complicated with various septic emboli and thrombosis of the internal jugular vein [7]. In addition, *F*. *necrophorum* is also associated with peritonsillar abscess formation and otitis media in small children [8]. Bacteremia due to other *Fusobacterium* spp. is uncommon and is associated with a variety of clinical presentations [9].

In addition, *Fusobacterium* spp. in the oral cavity are carcinogenic and are considered as a risk factor for pancreatic and colorectal cancers [10]. They have recently been implicated in a broad spectrum of human pathologies, including Crohn’s disease, ulcerative colitis (UC), preterm birth, and colorectal cancer [11]. Some *Fusobacterium* spp. are capable of actively invading host cells without the aid of other factors; the active invader species *F*. *nucleatum* and *F*. *periodonticum* can independently invade host cells in part using extracellular adhesin and invasion molecules, such as *Fusobacterium* adhesion (FadA) [12,13]. Other *Fusobacterium* spp. are passive invaders, including *F*. *necrophorum*, a veterinary pathogen and causative agent for necrosis; and *F*. *ulcerans*, which is related to tropical skin ulcers. *F*. *mortiferum* and *F*. *varium* are frequent residents of the human gut [14]; some experimental data suggest that *F*. *varium* can actively invade host epithelial cells [14].

In this study, we analyzed the whole-genome sequence of *F*. *varium* Fv113-g1 isolated from a patient with UC. Furthermore, a comparative analysis suggested that Fv113-g1 possesses many redundant paralogs of *Fusobacterium* virulence factors, including autotransporters (type V secretion system, T5SS), FadA, and hemaggulutinin.

## Materials and methods

### Ethics statement

The study protocol was approved by the institutional medical ethics committee of the National Institute of Infectious Diseases in Japan (Approval No. 479 and 642) and conducted according to the Declaration of Helsinki guidelines. Before molecular diagnosis for etiological pathogens, a written informed consent was obtained from the patient to isolate potential etiological agents.

### Bacterial strain

*F*. *varium* Fv113-g1 was isolated from the colon mucus membrane of a patient with UC [15]. Briefly, the mucus biopsy specimens were first incubated with imipenem (50 μg/ml saline) for 1 hour at 37° C to inactivate extracellular bacteria organisms, and then serially diluted samples were spread on agar plates for anaerobic culture to isolate intracellular bacteria in colon mucus epithelium. Isolated bacteria were examined by the disk diffusion test for imipenem susceptibility (minimal inhibitory concentration less than 4 μg/ml). Fv113-g1 was cultivated in modified GAM broth (Nissui Pharmaceutical Co., Ltd., Tokyo, Japan) or on FM agar (Becton Dickinson, New Jersey, USA) under anaerobic conditions at 37°C.

On the basis of the ATCC product sheets, no sufficient information of pathogenicity for ATCC 8501 and 27725 have been stated; in addition, those isolates are categorized as biosafety level 1, suggesting that those are commensal bacteria as one of human gut flora.

### Pulsed-field gel electrophoresis

Pulsed-field gel electrophoresis (PFGE) plug was prepared using the CHEF Bacterial Genomic DNA Plug Kit (BioRad, CA, USA), replacing lysozyme with achromopeptidase (Wako, Osaka, Japan) for bacterial lysis. The plug was treated with a restriction enzyme or S1 nuclease, followed by PFGE (1% agarose gel; 0.5× TBE; 6 V/cm; pulse-time, 2.2–65.0; angle, 120°; run time, 20 h) [16].

### Whole-genome sequence analysis

Detail experimental procedures for hybrid assembly using illumina short reads, PacBio long reads and Argus optical mapping were illustrated in S1 Fig. Genomic DNA of *F*. *varium* Fv113-g1 was purified as follows: bacterial cells were lysed with achromopeptidase (Wako), followed by phenol–chloroform extraction and further purification using the Qiagen DNA purification kit (Qiagen, Germany). Short insert size (approximately 1.4 kb) for paired-end and long insert sizes (3–4 kb, 5–7 kb, and 8–10 kb) for mate-pair library were constructed using the Illumina Nextera XT DNA Sample Prep Kit (Illumina, San Diego, CA, USA) and Nextera Mate-Pair Sample Prep kit (Illumina), respectively. Whole-genome sequencing of Fv113-g1 was performed using the Illumina MiSeq platform with 300-cycle MiSeq Reagent Kit v2 for the paired-end library and 600-cycle MiSeq Reagent Kit v3 for the mate-pair libraries. The short reads were assembled using Platanus v1.0.1 [17] and A5-Miseq *de novo* assembler [18]. The complete genome sequence of this strain was determined using the PacBio RSII sequencer for long-read sequencing method (DNA/Polymerase Binding Kit P5; DNA Template Prep Kit 3.0; insert size, approximately 20 kb). Sequencing data were produced with more than 30-fold coverage and assembled using the assembly program SMRT 2.3.0_HGAP3 [19]. Argus optical mapping with *NcoI* of Fv113-g1 genome DNA was performed using the Argus system (OpGen, MD, USA) according to the manufacturer’s protocol and previous report by Hasegawa *et al*. [20]. Error correction of tentative complete circular sequences was performed using iCORN2 in PAGIT version 1.64 [21] with Illumina short reads. Annotation was performed in Rapid Annotation using Subsystems Technology 2.0 (RAST) [22], InterPro v49.0 [23], and NCBI-BLASTp/BLASTx.

### Comparative genome sequence analysis

The draft-genome sequencing of *F*. *varium* type strain ATCC 8501^T^ was performed as described in the abovementioned section, followed by *de novo* assembly using A5-Miseq *de novo* assembler [18]. Comparative genome analysis was performed with TBLASTx search (≥90% amino acid identity) using open reading frames (ORFs) of Fv113-g1 as queries against draft-genome sequences of strains ATCC 8501^T^ and ATCC 27725, ATCC 49185 and 12-1B, respectively (S1 Table), followed by visualizing the search results with GView server (https://server.gview.ca/) [24].

Phylogeny was generated using the maximum-likelihood phylogenetic method with FastTree v2.1.10 [25].

Orthology analysis was performed using OrthoVenn that is a web platform for comparison and annotation of orthologous gene clusters among multiple species (threshold: <e^-10^) [26].

### RNA-seq analysis

Bacterial cells were suspended in 10 mM Tris-10 mM EDTA (TE_10_ buffer), 1% Sodium dodecyl sulfate, and phenol–chloroform and then subjected to extraction. The upper phase of the extraction was subjected to RNA purification using the RecoverAll^TM^ Total Nucleic Acid Isolation Kit (Life Technologies) according to the manufacturer’s instructions. RNA-seq libraries were prepared from approximately 30 ng of total RNA using the ScriptSeq^TM^ v2 RNA-Seq Library Preparation Kit (Epicentre Biotechnologies) according to the manufacturer’s instructions. The RNA-seq libraries were sequenced as single-end 151-mers on a MiSeq sequencer using the MiSeq Reagent Kit v3 (Illumina). The transcriptome analysis was performed using CLC Genomics Workbench 7.5 software (Qiagen K.K.). Significant gene expression was determined using a false discovery rate-normalized *P* value of <0.05. All RNA-seq raw data are available in S2 Table.

### Nucleotide sequence accession number

The whole-genome sequence and annotation are available in GenBank: FV113-g1-chromosome (AP017968), pFV113-g1-1 (AP017969), pFV113-g1-2 (AP017970); BioProject PRJDB5491. The short-read sequences for DNA-Seq and RNA-Seq have been deposited in DNA Data Bank of Japan (DDBJ; accession numbers: DRA005489 and DRA005507). In addition, draft-genome sequence of reference type strain ATCC 8501^T^ has been also deposited in DNA Data Bank of Japan (DDBJ; accession numbers: DRA006297).

## Results and discussion

### Complete genome sequencing of *F*. *Varium* FV113-g1

First, the draft-genome sequencing of *F*. *varium* Fv113-g1 was obtained with paired-end and mate-pair short-read libraries. The total number of assembled scaffolds was 3417, with a total scaffold length of approximately 4.4 Mb. N50 and the longest scaffold were approximately 242 kb and 858 kb in length, respectively. The draft-genome sequence constituted many gaps (regions of Ns) in assembly scaffolds; therefore, the short-read assembly only was not efficient to determine the complete genome sequence of Fv113-g1. To complete the genome sequences, PacBio long-read sequencing was performed, resulting in five unitigs (total, 4.14 Mb; N50, 3 Mb). To fill the remaining gaps in these five unitigs, optical mapping of the whole genome digested with *NcoI* restriction enzyme was performed, followed by gap filling and circularization with nucleotide alignment and error correction with iCORN.

**Fig 1:**
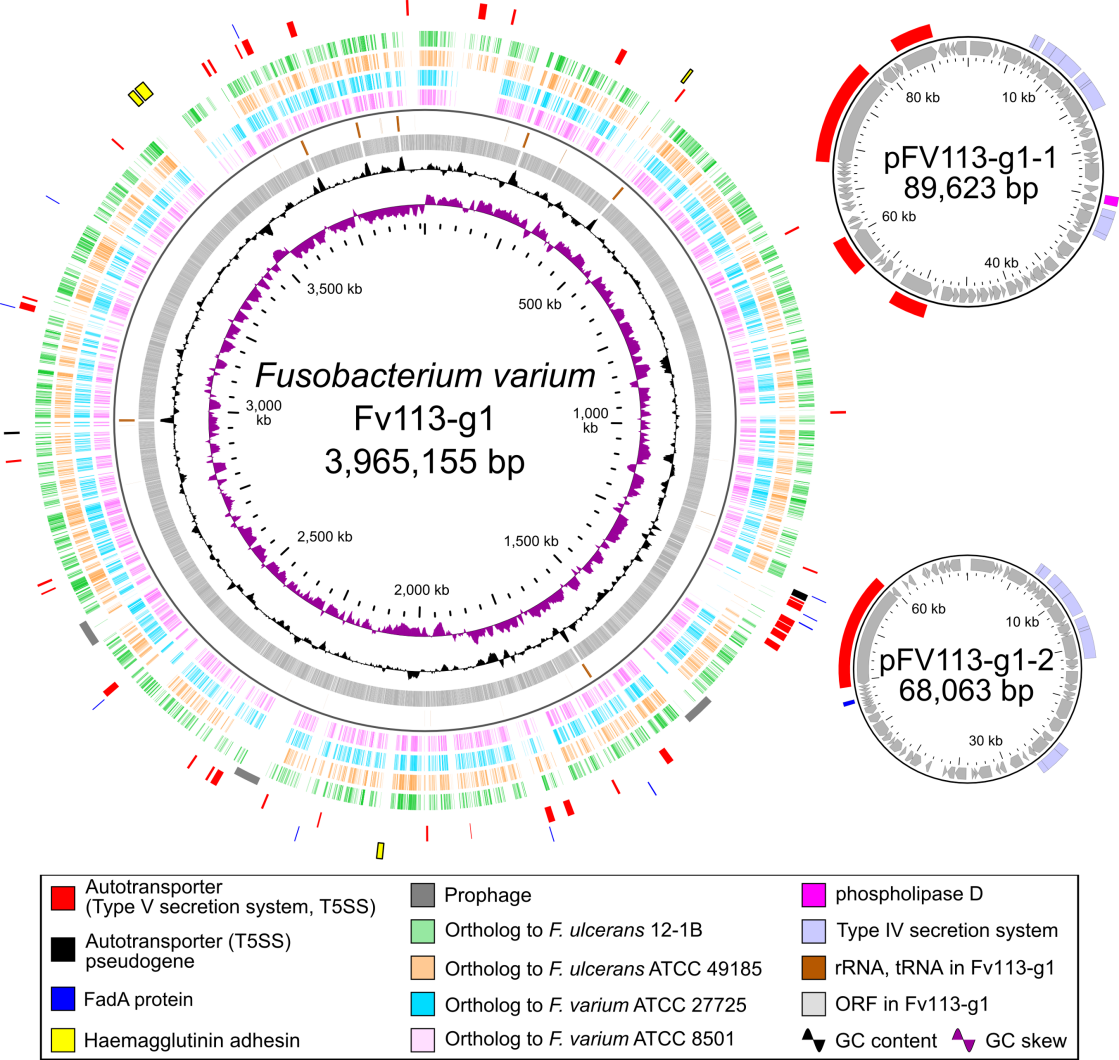
Basic genome information of *F*. *varium* Fv113-g1. Circular representation of the Fv113-g1 genome (chromosome and two plasmids) along with comparative genome information of other *F*. *varium* strains (S1 Table). Fv113-g1 genomic information is shown. From inward, slots 1–4 (slot 1, GC skew; slot 2, GC content; slot 3, open reading frames; slot 4, RNAs), slots 5–8 (comparative genome analysis of ATCC 8501^T^, ATCC 27725, ATCC 49185 and 12-1B, respectively, with ≥90% aa identity), slot 9 (prophage), and slots 10–11 (possible virulence factors: autotransporter, FadA protein, and hemagglutinin).

**Fig 2:**
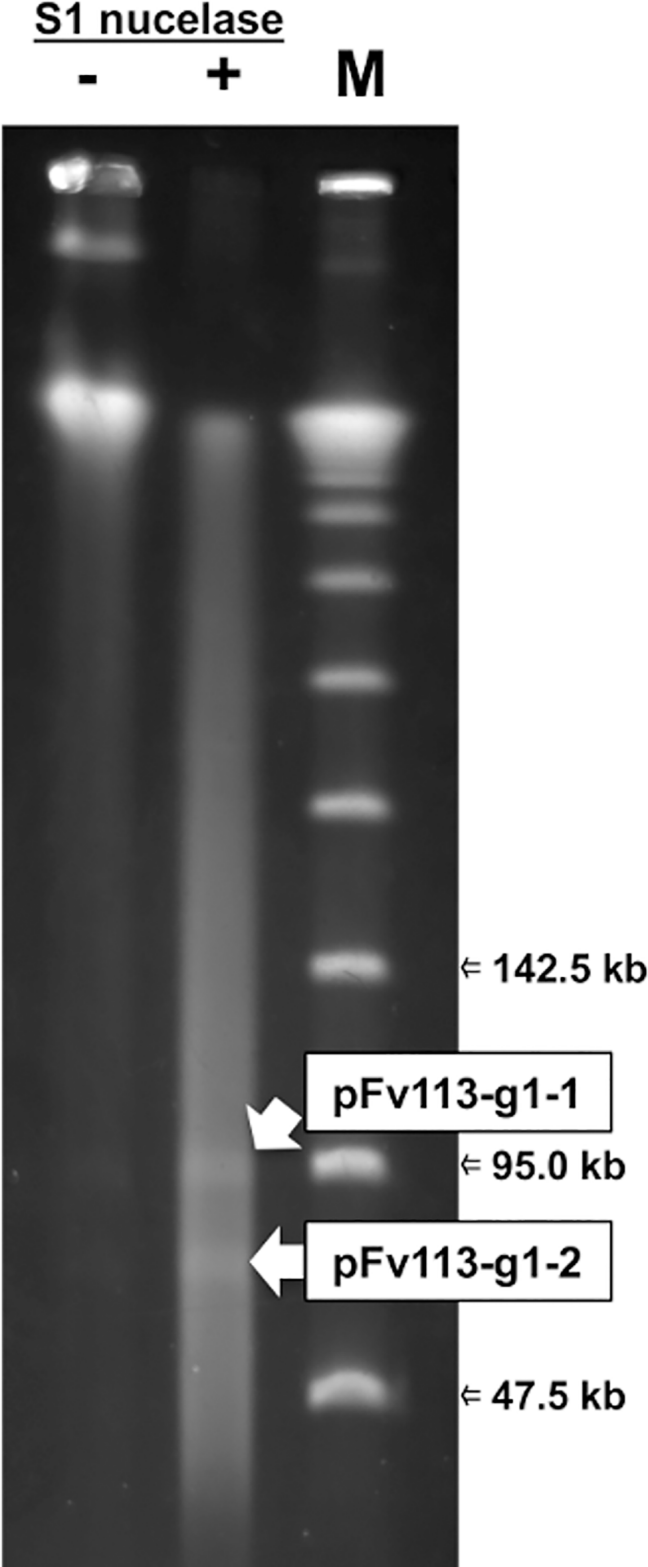
Two plasmids in Fv113-g1. Plasmids were identified by separation on Pulsed-field gel electrophoresis using S1 nuclease-treated genomic DNA.

The complete genome sequence suggested that Fv113-g1 possesses one chromosome and two plasmids (pFV113-g1-1 and pFv113-g1-2) as shown in Fig 1. These chromosome and plasmid sequences were verified by PFGE using the restriction enzyme *AscI* (data not shown) and S1 nuclease-treated DNA (Fig 2). Moreover, putative two plasmid bands were extracted from the PFGE gel, followed by sequencing with Illumina MiSeq, assembling with A5-Miseq, resulting in complete plasmid sequences. Two complete plasmid sequences were also finished by circularization and error correction as described above.

The final Fv113-g1 genome information is shown in Table 1. The chromosomal DNA was 3.96 Mb long, encoding 58 tRNA genes, seven rRNA operons, 3,552 predicted coding sequences, and seven pseudogenes (Table 1). The GC content of the chromosomal DNA was 29.2%, whereas that of plasmids was 26.7% and 27.7%, respectively, indicating relatively low GC content (Table 1).

**Table 1:** Genomic information of *F*. *varium* Fv113-g1.

	Chromosomal DNA	Plasmid DNA	Plasmid DNA
	Fv113-g1	pFv113-g1-1	pFv113-g1-2
Length (bp)	3,965,155	89,623	68,063
GC%	29.2	26.7	27.7
CDS	3,552	78	63
Average length CDS (nt)	983.55	982.08	901.48
Coding%	89.0%	85.5%	83.4%
rRNA	22	0	0
tRNA	58	0	0
Genbank ID	AP017968	AP017969	AP017970

### Insertion sequence (IS) in *F*. *varium* Fv113-g1

Multiple copies of two ISs designated as IS*Fv1* (1.44 kb) and IS*Fv2* (1.78 kb) were identified as 47 and 48 insertions in the chromosome, respectively (blue and green bar in the most outer circle in Fig 1). The coding sequence of IS*Fv1* was relatively similar to that of IS*91* family and orthologs of *F*. *varium*/*necrophorum* strains, but with very low similarity of up to 85%. However, it was similar to the IS*91* family in *Leptotrichia goodfellowii* or *Streptobacillus moniliformis*, which are members of the family Leptotrichiaceae, suggesting that these IS*Fv1* orthologs might have possibly disseminated with horizontal gene transfer between Fusobacteriaceae and Leptotrichiaceae. On the contrary, the coding sequence of IS*Fv2* showed high similarity to IS*5*/*1182* family and widely identified orthologs of *F*. *nucleatum* strains, suggesting that IS*Fv2* could be a general orthologous IS among *Fusobacterium* spp.

### Comparative genomics among *F*. *varium* genome sequences

To characterize the Fv113-g1-specific genetic features, we additionally sequenced the draft-genome sequence of *F*. *varium* ATCC 8501^T^ (total, 3.3 Mb) as one of the reference genomes, followed by comparative genome analysis of *F*. *varium* ATCC 8501 ^T^ and publicly available draft-genome sequence of ATCC 27725 (3.3 Mb; NZ_ACIE00000000.2). ATCC 8501^T^ and ATCC 27725 are categorized as biosafety level 1, suggesting that those are commensal bacteria as one of human gut flora. The chromosomal DNA of Fv113-g1 was larger by approximately 0.66 Mb than that of ATCC 8501^T^ and ATCC 27725 (S1 Table).

**Fig 3:**
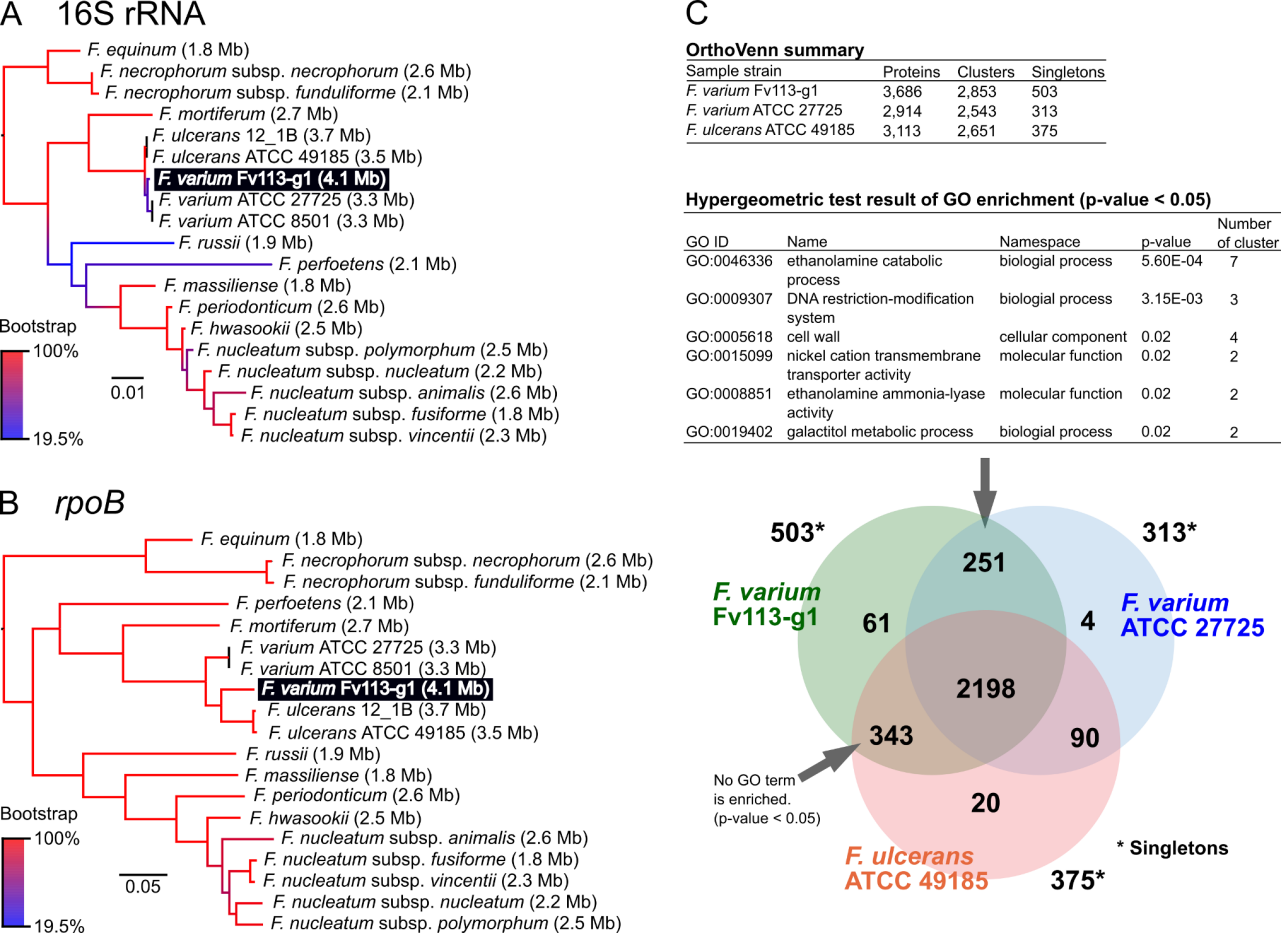
Phylogenetic and orthology analysis among Fv113-g1 related *Fusobacterium* spp. Maximum-likelihood phylogenetic analysis with 1000× bootstrapping was performed for **A)** 16S-rRNA (1,370 nt) and **B)** RNA polymerase β-subunit gene *rpoB* among *Fusobacterium* spp. listed in S1 Table. **C)** Venn-diagram of orthology analysis among indicated three strains (threshold: <e^-10^). OrthoVenn website generated gene clusters as ortholog in every strain, for instance, all predicted proteins in Fv113-g1 (3686 proteins) were discriminated into 2853 clusters, whereas the remaining proteins (503 proteins) were singletons showing no orthologous proteins.

Phylogenetic analysis using 16S-rRNA among 18 *Fusobacterium* species (S1 Table) indicated that Fv113-g1 appears to be basically classified as *F*. *varium* (Fig 3A), whereas further phylogenetic analysis for the *rpoB* gene displayed the relatively discriminated phylogeny between those two (Fig 3B). In addition, orthology analysis indicated that 65.5% [2,198 / (2,853 all clusters + 503 singletons) in Fv113-g1] of gene clusters are identified as core clusters among *F*. *varium* related strains, whereas 343 orthologous gene clusters between Fv113-g1 and *F*. *ulcerans* ATCC 49185 found to be more than 251 clusters between Fv113-g1 and *F*. *varium* ATCC 27725 (Fig 3C and S2 Table). Orthologous analysis also suggested that Fv113-g1 appears to possess both genetic features of *F*. *varium* and *F*. *ulcerans*. Indeed, pan-genome analysis suggested that Fv113-g1 possesses notable Fv113-g1-specific genetic features as follows: redundant T5SS (red bar in Fig 1); FadA paralogs (blue bar in Fig 1); two large, filamentous hemagglutinin adhesions (yellow bar in Fig 1; FV113-G1_31500, 3,125 aa; FV113-G1_31550, 5,066 aa); multiple ISs, and three potential prophages (gray bar in Fig 1) located on the chromosome. Abovementioned comparative genome analyses totally suggested that Fv113-g1 should be basically assigned as *F*. *varium*, in particular, it could be reclassified as notable *F*. *varium* subsp. similar to *F*. *ulcerans* because of partial shared orthologs.

### Autotransporters T5SS in *F*. *varium* Fv113-g1

**Fig 4:**
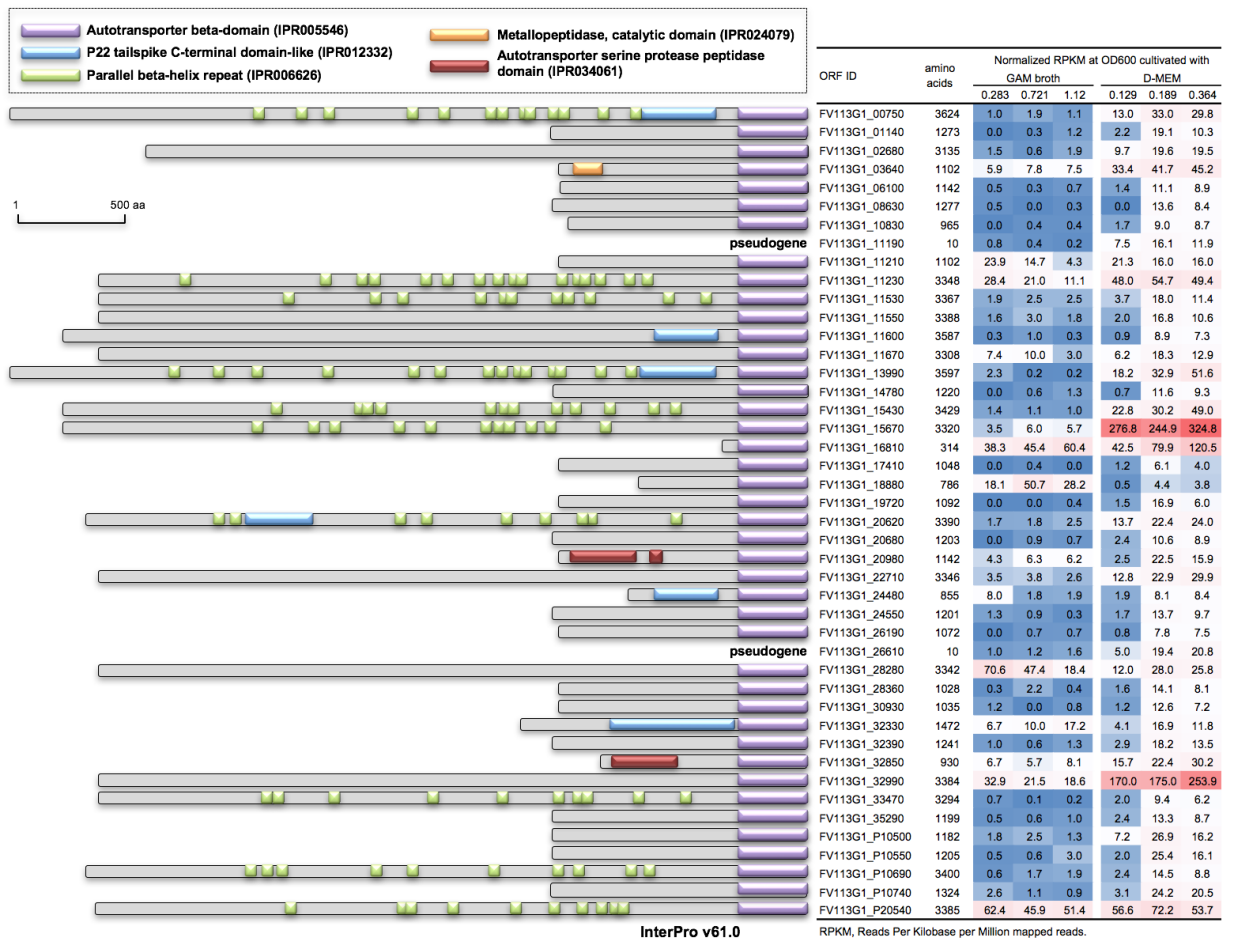
Autotransporters as type V secretion system (T5SS) in *F*. *varium* Fv113-g1. A total of 39 T5SSs, including two pseudogenes, are located on the chromosome and four and one in plasmids pFV113-G1-1 and pFv113-g1-g1-2, respectively. T5SS possesses the presumed integral transmembrane β-barrel domain (IPR005546) at the C-terminus, which mediates its secretion through the outer membrane to transport the protein itself. Comparative transcriptome analysis was performed using RNA-seq under different media (GAM broth or D-MEM) and growth phase conditions (see differential expression of all ORFs in S2 Table). The expressions of two T5SSs (FV113-G1_15670 and FV113-G1_32990) were found to be significantly increased in D-MEM, and six T5SSs showed relatively high expressions.

Fv113-g1 possesses the multiple T5SS, which have been characterized as autotransporters. Also, 39 T5SSs, including two pseudogenes, were located on the chromosome and four and one in plasmids pFV113-g1-1 and pFv113-g1-2, respectively (Fig 1). T5SS was first characterized for IgA1 protease [27]; the presumed integral transmembrane β-barrel domain (IPR005546) at the C-terminus mediates its secretion through the outer membrane to transport the protein itself (Fig 4). The N-terminus contains the variable passenger domain, resulting in autocatalytic cleavage in some proteins, whereas a different protease is used in other proteins. However, no cleavage occurs in some cases [28]. Some N-terminus domains showed typical parallel β-helix repeats (InterPro ID: IPR00626), which hindered the gap closing during *de novo* assembly of the whole-genome sequence (Fig 4). Two possible serine proteases FV113-G1_20980 and FV113-G1_32850 and one possible metallopeptidase FV113-G1_03640 were predicted by InterPro motif search, but most others showed unknown function, suggesting that such multiple T5SSs have not been well characterized for the potential virulence of Fv113-g1. However, Fv113-g1 carries the most redundant T5SS compared with other *Fusobacterium* spp. and bacteria (Table 2), which also implies that Fv113-g1 has a high potential of surviving in hostile environments as a virulent strain.

**Table 2:** Predicted number of autotransporters and FadA adhesions in the whole-genome sequence of *Fusobacterium* spp.

Bacteria species	Strain	Number of predicted autotransporters	FadA adhesions	Reference
*Fusobacterium varium*	Fv113-g1	44	13	This study
*Fusobacterium varium*	ATCC 27725	32	5	InterPro
*Fusobacterium ulcerans*	ATCC 49185	32	8	InterPro
*Fusobacterium ulcerans*	12-1B	32	7	InterPro
*Fusobacterium nucleatum*	13_3C	8	4	InterPro
*Fusobacterium nucleatum*	CTI-1	15	4	InterPro
*Fusobacterium nucleatum*	CTI-5	11	3	InterPro
*Fusobacterium nucleatum*	CTI-6	6	1	InterPro
*Fusobacterium nucleatum* subsp. *animalis*	11_3_2	17	5	InterPro
*Fusobacterium nucleatum* subsp. *animalis*	4_8	15	4	InterPro
*Fusobacterium nucleatum* subsp. *animalis*	7_1	15	4	InterPro
*Fusobacterium nucleatum* subsp. *animalis*	D11	15	4	InterPro
*Fusobacterium nucleatum* subsp. *animalis*	F0419	17	4	InterPro
*Fusobacterium nucleatum* subsp. *nucleatum*	ATCC 23726	12	3	InterPro
*Fusobacterium nucleatum* subsp. *nucleatum*	ATCC 25586	15	3	InterPro
*Fusobacterium nucleatum* subsp. *polymorphum*	ATCC 10953	10	4	InterPro
*Fusobacterium nucleatum* subsp. *vincentii*	3_1_36A2	13	3	InterPro
*Fusobacterium nucleatum* subsp. *vincentii*	4_1_13	13	5	InterPro
*Fusobacterium nucleatum* subsp. *vincentii*	ATCC 49256	14	2	InterPro
*Fusobacterium periodonticum*	1_1_41FAA	15	4	InterPro
*Fusobacterium periodonticum*	2_1_31	19	4	InterPro
*Fusobacterium periodonticum*	ATCC 33693	22	5	InterPro
*Fusobacterium periodonticum*	D10	18	5	InterPro
*Fusobacterium* spp.	CM1	10	2	InterPro
*Fusobacterium* spp.	CM21	15	3	InterPro
*Fusobacterium* spp.	CM22	8	3	InterPro
*Fusobacterium* spp.	OBRC1	10	4	InterPro

InterPro database suggested the number of predicted autotransporters showing the presumed integral transmembrane ß-barrel domain (IPR005546) at the C-terminus, mediating its secretion through the outer membrane to transport the protein itself (Fig 3) (http://www.ebi.ac.uk/interpro/entry/IPR005546/taxonomy). Fusobacterium adhesion FadA homologs (IPR018543) are also identified. (http://www.ebi.ac.uk/interpro/entry/IPR018543/taxonomy)

### FadA paralogs in *F*. *varium* Fv113-g1

Some *Fusobacterium* spp. are capable of actively invading host cells using extracellular adhesin and invasion molecules, such as FadA [12,13]. Analysis of FadA homologs among *Fusobacterium* spp. suggested that most species carry an average of four FadA homologs, whereas *F*. *varium* Fv113-g1 carries 13 (Fig 1 and Table 2), implying that significantly redundant FadA paralogs could contribute to severe mucosal inflammation, which might lead to UC. In fact, it has been reported that *F*. *nucleatum* FadA binds to E-cadherin, thus activating β-catenin signaling and differentially regulating inflammatory and oncogenic responses [29]. Moreover, in xenografts, FadA treatment alone stimulates the inflammatory responses, indicating that FadA is a major stimulant of inflammation.

**Fig 5:**
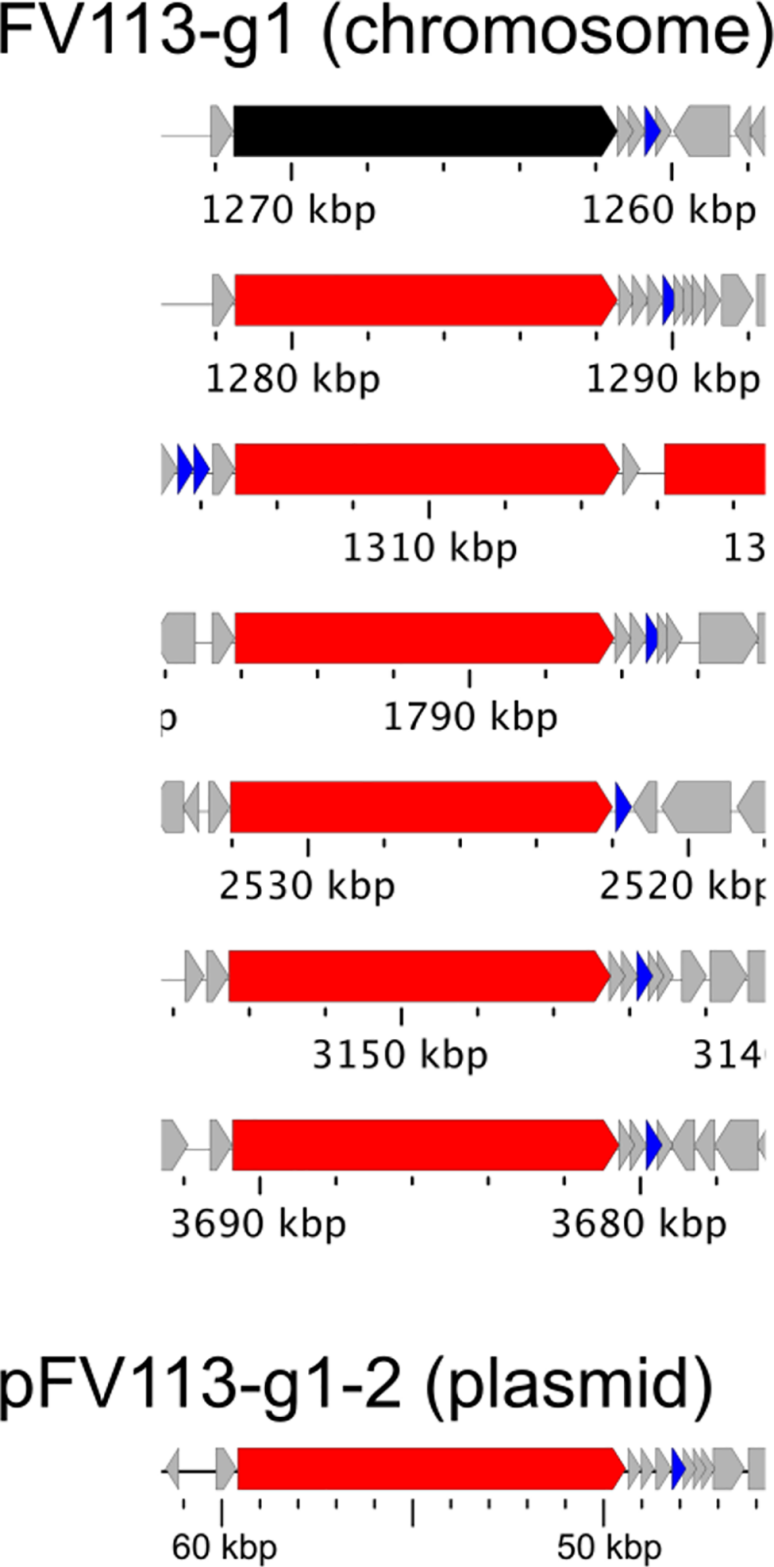
FadA paralogs adjacent to T5SS. Potential invasion molecule FadA is closely located to T5SS, possibly associated with Fv113-g1 pathogenicity. ORF color is also illustrated in Fig 1 (red, T5SS; black, pseudo T5SS; blue, FadA paralogs).

Intriguingly, the paralogs of potential invasion molecule FadA and T5SS are closely located (Fig 5), implying that FadA-related pathogenicity of Fv113-g1 may be coordinately associated with multiple T5SS.

### Filamentous hemagglutinin in *F*. *varium* Fv113-g1

Two large potential adhesions (FV113-G1_31500, 3,125 aa, 328 kDa; FV113-G1_31550, 5,066 aa, 563 kDa) were found as Fv113-g1-specific surface proteins, which showed similarity to a protein motif corresponding to N-terminal filamentous hemagglutinin (InterPro ID: IPR008638). Blastp homology search suggested that there are no orthologous proteins for these adhesions and showed more than 30% aa similarity to hemagglutinin in *F*. *necrophorum* strains. A number of filamentous hemagglutinins, including many proteins of more than 2500 amino acids, represent a carbohydrate-dependent hemagglutination activity domain found near the N-terminus [30].

### Comparative transcriptome analysis between BHI and D-MEM

We speculated that cell culture medium D-MEM could reproduce *in vivo* conditions to some extent for the bacteria instead of a nutrient-rich broth, such as BHI. To identify potential virulence-related factors showing increased gene expression in D-MEM, we performed comparative transcriptome analysis using RNA-seq under different media and growth phase conditions. In total, 107 ORFs were identified (S3 Table); the expression of putative transport systems, mainly iron and other cations of the ABC transporter, was significantly increased, suggesting that a difference in the culture medium apparently affects the acquisition of ferrous ions. In addition, the expression of ferric enterobactin receptors was increased. Such differential expression could be because of limited nutrient supply by D-MEM because metal acquisition is vital for bacteria growing in metal-scarce environments, such as inside a host in the presence of pathogens.

The most significant high expression was found in flavodoxin FldA ortholog (FV113-G1_04600). Flavodoxin is a small redox-active protein with a flavin mononucleotide prosthetic group. It functions as an electron transfer agent in a variety of microbial metabolic processes, including nitrogen fixation by nitrogenase [31] and sulphite reduction [32]. A putative alkyl hydroperoxide reductase (FV113-G1_28520), a peroxiredoxin belonging to a family of ubiquitous proteins that are important for defense against antioxidants, was highly overexpressed in D-MEM culture [33]. These findings suggested that a medium shift may affect the metabolic pathway underlying the oxidative stress generated by D-MEM-dependent energy production. Flavodoxin and peroxiredoxin are potential virulence-related factors, because they contribute to protect the bacterial cell from oxidative stress in the phagolysosome of the macrophage to thrive in hostile environments [34]; such antioxidants have been shown to be essential in several other bacteria (http://tubic.tju.edu.cn/deg/) [35]. In addition, flavodoxin is an essential factor for the survival of some human pathogens, and the fact that flavodoxin is not present in humans strongly suggests the possibility of drug development of novel and specific antimicrobial agents against multidrug-resistant bacteria [36].

The expression of two T5SSs (FV113-G1_15670 and FV113-G1_32990) significantly increased in D-MEM, and six T5SSs showed relatively higher expressions (Fig 4). As described above, the specific features of T5SS have not been well characterized, but medium-dependent expression may contribute to Fv113-g1 survival as well as the abovementioned flavodoxin-related metabolism.

Of the 13 FadA homologs (Table 2), two (FV113-G1_15700 and 32960) showed more than 40-fold and more than 10-fold increased expression in D-MEM, respectively; these ORFs were closely located to the two T5SSs discussed above, suggesting that FadA and T5SS are coordinately modulated to adapt to the environmental change. One homolog (FV113-G1_19130) showed 15-fold reduced expression (S2 Table), whereas two other homologs (FV113-G1_14340 and _14350) showed highly constitutive expression. RNA-seq experiment suggested that these redundant FadA homolog genes are differentially regulated to support the well-growing bacteria under variable conditions.

## Conclusions

We determined the complete genome sequence of *F*. *varium* Fv113-g1 isolated from a patient with UC. Comparative genome analysis revealed that Fv113-g1 possesses noteworthy gene repertories, including the most redundant T5SS and FadA adhesins, in both the chromosome and plasmids. The genome size of Fv113-g1 is apparently larger than that of ATCC-type strains, and transcriptome analysis suggested that Fv113-g1-specific accessary genes, such as multiple T5SS, could potentially contribute to survival against other human gut microbiota and to the pathogenicity to human intestinal epithelium.

## Supporting information

S1 Fig Experimental procedures for complete genome sequence of Fv113-g1. (PDF)

S1 Table List of *Fusobacterium* spp. for comparative genome analysis. (XLSX)

S2 Table Orthology and RNA-seq results of *F*. *varium* Fv113-g1 with differential media and growth phase conditions. (XLSX)

S3 Table Potential virulence-related factors showing increased gene expression in D-MEM. (XLSX)
