# Supplementary figures and images for: Characterization of Fusobacterium varium Fv113-g1 isolated from a patient with ulcerative colitis based on complete genome sequence and transcriptome analysis
Source: PLoS One. 2017 Dec 7;12(12):e0189319. doi: 10.1371/journal.pone.0189319 (PMC5720691; doi:10.1371/journal.pone.0189319)

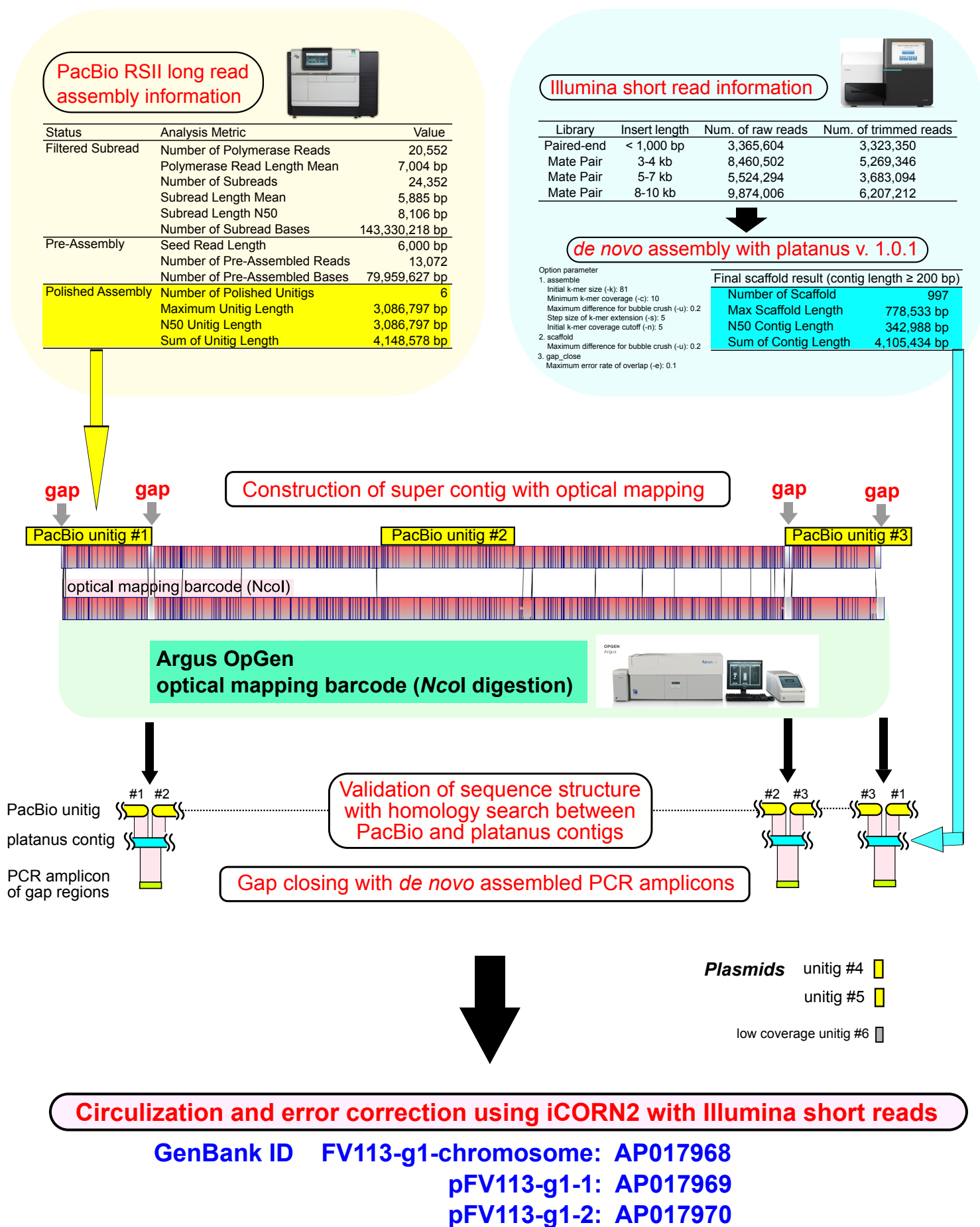

**Fig. S1. Experimental procedures for complete genome sequence of Fv113-g1**

Supplement: S1 Fig — (PDF) [file pone.0189319.s001.pdf]
